# Motivations and Personal Traits Can Predict Self-Efficacy of the Clown Therapist: A Descriptive Study

**DOI:** 10.3390/ijerph19127058

**Published:** 2022-06-09

**Authors:** Pierluigi Diotaiuti, Stefania Mancone, Stefano Corrado

**Affiliations:** Department of Human Sciences, Society and Health, University of Cassino and Southern Lazio, 03043 Cassino, Italy; s.mancone@unicas.it (S.M.); stefano.corrado@unicas.it (S.C.)

**Keywords:** clown therapist, personality, motivation, self-efficacy, teamwork

## Abstract

The individual and collective perception of self-efficacy in clown therapists is fundamental in order to be able to be active, restrained, energetic and defocused, if necessary, without being overwhelmed by any problems patients might have. The present study evaluated both the incidence of motivational and dispositional functions on the level of perceived self-efficacy with a sample of 259 Italian clown therapists who were administered *The Volunteer Self-Efficacy Scale*, the *Volunteer Process Model* and the *Italy Personality Inventory*. The significance of a hierarchical linear regression model of perceived self-efficacy was tested. The results showed that the value orientation of the operators mainly influenced the level of perceived self-efficacy, that is, the search for actions with a high social meaning, rather than the orientation towards situations and experiences that allow one to expand one’s knowledge and promote one’s own person. Profiles with higher perceived self-efficacy were associated with the trait of dynamism and conscientiousness, while a person’s vulnerability was found to be a significant negative predictor of self-efficacy. An additional significant predictor was the experience of the clown therapist. The results of the study also showed a positive and functional role of the synergy conferred by teamwork. The group mitigates the emotional difficulties of the individual and supports him/her by orienting him/her technically and compensating for any inexperience in the field of animation in sensitive contexts, such as hospital wards with serious and vulnerable patients, such as children.

## 1. Introduction

Clown therapy represents a special way of using humor to promote people’s well-being. Clown therapy officially began in 1986 and it is currently easy to come across clown doctors in hospitals. Clown therapy is defined as the application of clown techniques derived from the circus world to contexts of illness, in order to improve people’s mood and state of mind [1]. The presence of professional clowns working in hospitals as part of the healthcare team dates back to thirty years ago. The birth date of clown therapy dates back to 1986, when Karen Ridd in Winnipeg and Michael Christensen in New York independent of each other began the practice of clowning in large children’s hospitals [2,3]. In Italy, the first Clown Care Unit, called Fondazione Theodora, was created in Milan in 1995 [4].

One of the most salient aspects related to clowning is humor. The purpose of clowning is to bring smiles and laughter to an audience of all ages. Contrary to popular opinion, clowns are not strictly children’s entertainers, as adults can enjoy clowns as well [5,6]. An effective clown makes people laugh, so humor is his main tool. He should be able to accept humor as an integral part of life, improve his personal sense of humor, listen and learn how other people recognize and use humor, and be prepared to respond to the humor of others. In pediatrics, humor is increasingly present in the hospital setting, and clowns are often employed based on the assumption that humor is associated with patients’ well-being. Clinical staff have noted that the main benefit of therapeutic humor is its distraction technique, which takes patients’ minds away from worries and pain related to their illness and the resulting depressed mood, thus promoting a healthy expression of emotions [7,8,9,10,11,12].

Clown therapy is implemented by people who are specially trained to operate in the social and health sector and belong to private entities (associations, cooperatives, foundations, etc.), who choose the clown as a state of consciousness to enter into a relationship with people in hospital (or in difficulty), using techniques derived from theatrical improvisation, the art of clowning and micro-prestidigitation [13,14].

Clown therapy interventions are aimed at the community of places of care (hospitals, nursing homes, family homes, orphanages, day care centers, reception centers) in order to alleviate the state of anxiety and suffering of patients (especially children) and, simultaneously, to improve the functionality of the immune system [15,16].

The literature on humor in hospital wards at different age levels shows that not only do patients and medical staff benefit from humor, but interactions involving humor between hospital staff and patients foster an atmosphere in which laughter and humor are self-perpetuating [17,18,19]. Other investigations report that humor has beneficial effects on stress related to terminal illness [20], pain tolerance [21], and cognitive functions, such as memory and anxiety [22,23,24].

Recent research conducted in Canada as part of the Down Memory Lane project suggests that senior clowns can help seniors improve communication skills, mood, and quality of life [25,26]. Senior clowns may also help some older people with dementia connect with their surroundings and restore a sense of independence to individuals who have little control over their lives. This activity is especially helpful for residents who do not receive many visitors. In addition, the presence of senior clowns can have a positive effect on the feelings of staff members who care for the elderly [27].

Smile therapists are actually widespread throughout Italy; grouped into four federations (Laughing for Living, National Federation of Clown Doctors, Nasi Rossi d’Abruzzo, and Vip-Viviamo in positivo), there are an estimated 3000 active volunteers who serve in over 100 hospitals and social-health facilities in Italy [28].

In the literature, the only previous study that has investigated the motivations of clown therapists was proposed by Strollo et al. [29]. Within this work, the identikit of a clown therapy volunteer is presented as a subject, mostly female, with an average age of 35 years and a high average level of education and economic status. As reiterated by Strollo et al. [29], those who engage in this type of service find it is essential to have the perception of self-efficacy, both individual and collective, namely the sense of having the adequate skills to handle difficulties and stress, to share their feelings with other members of the association and, at the same time, to empathize with others. Recently, the study by Dionigi et al. [30] highlighted the association between perceived self-efficacy and the psychological well-being of volunteer clown doctors. Here, results seem to confirm the theoretical hypothesis according to which only a person who is able to immerse himself emotionally in a situation but also to control his moods, without getting lost in his total identification with the person in need, will then be able to effectively carry out his tasks [31]. 

In 2016, Dionigi proposed a study comparing the personality traits of a sample of Italian clown doctors (volunteers and professionals) with those of the normal population. The study was prompted by the intention to understand whether clown doctors possess specific personality traits related to fundamental aspects of such a practice [32]. The clown doctors’ scores were higher in extraversion, agreeableness, conscientiousness, and openness to experience, and lower in neuroticism compared to published normative data. 

Other works in the literature have shown that coping strategies in volunteers and the quality of the relationships established with other members of the facility where they serve are significant moderating factors in hospital volunteer drop-out caused especially by emotional exhaustion, personal distress, and high levels of burn-out [33,34,35,36]. Social cognitive factors, such as self-efficacy and motivation, demonstrated strong effects in behavior intention under challenging environments, such as those in hospitals that constantly confront the volunteer with the suffering, anxieties, and heavy limitations of patients [37,38,39,40]. Generally, self-efficacy and motivation are considered antecedents of perceived involvement [41,42], but according to others, the reverse model would also be sustainable: experience, motivation, and perceived involvement would go a long way toward increasing the hospital worker’s sense of self-efficacy by promoting a state of greater mental and physical well-being [43,44]. Again in the hospital setting, further interesting studies have shown in volunteer samples the importance of perceived self-efficacy in limiting stress and improving the perceived quality of life [45,46].

Compared to previous studies by Strollo et al. [29] and Dionigi [30], our contribution intended to assess the effects of both the motivational and dispositional functions on the level of perceived self-efficacy. Therefore, our research question was to test if among the predictors of self-efficacy perceived by a sample of clown therapy workers, personality traits and motivational functions could also play a significant role, in addition to socio-demographic characteristics. The goal was to more accurately identify the traits and the motivations associated with a higher level of perceived self-efficacy (positive predictors) and those associated with a lower level of perceived self-efficacy (negative predictors).

Considering the specificity of the intervention of the clown therapist, who usually works in an animation capacity in hospital settings where there is a need for emotional contact with patients (usually children) experiencing severe limitation and deprivation, it is plausible to believe that active and creative personal dispositions, together with general ideal motivations, are elements that enable them to perform this task best, while for those who have more introverted and defensive traits, and who are initially driven by more pragmatic reasons to such activities, it may be more difficult to measure up to the difficulties encountered in the field, and consequently they will feel more of an inadequacy or ineffectiveness in the role, and possibly feel the need to abandon such activity over time. 

More specifically, we identified the following hypotheses regarding predictors of self-efficacy in clown therapists, to be tested by statistical regression analysis: considering personality traits, based on the ITAPI model [47], we hypothesized to find the following traits among the positive predictors: dynamism, conscientiousness, empathy, and imagination; while for the negative predictors: introversion, vulnerability, and defensiveness. Regarding motivation, with reference to Omoto and Snyder’s model [48], we hypothesized to find value orientation, desire for personal growth, desire for relational contacts among the positive predictors and utilitarian motivations, desire to acquire knowledge and skills, desire for protection among the negative predictors. We considered the selection of negative motivational predictors of self-efficacy to be justified by the likely frustration induced by an emotionally oppressive environment with respect to any expectations of immediate pragmatic personal utility. Among the socio-demographic variables, we also hypothesized that greater accumulated experience in clown therapy might be an additional positive predictor of perceived self-efficacy. Finally, we assumed that in the identification of predictors of self-efficacy, variables related to the target of the intervention (children or the elderly) and the mode of conducting the intervention (in pairs or in a team) could have an influence, and therefore should also be considered in the analysis. As a sub-hypothesis associated with the service target, we considered that for working with children, the trait of dynamism of the clown therapist would be significant, while for working with the elderly, those with greater empathic ability would be more facilitated and therefore more gratified. Regarding the mode of conduct, we hypothesized that for those who worked in pairs, individual dynamism was an essential trait for the success of the intervention and also for the level of perceived self-efficacy. For those who carried out their service within a team, we felt instead that the trait of conscientiousness, that is, the ability to integrate well into the work group while respecting the agreed schedules, tasks and roles, was essential.

## 2. Materials and Methods

### 2.1. Participants and Procedure

The collection of information took place through the telematic administration of a protocol built ad hoc and associated with the Questbase platform. The protocol was emailed to representatives of the four Italian federations of clown therapists (Laughing for Living, National Federation of Clown Doctors, Nasi Rossi d’Abruzzo, and Vip-Viviamo in positivo), with a request to involve the members of the relevant confederate associations in the study. The members of these associations are not physicians but volunteers in the wards who have attended specific training courses in clown therapy, completed an average of 100 h of internship, including training and services in the hospital, carried out together with experienced clowns. Prior to completion, participants explicitly indicated their consent to aggregate data processing for research purposes. After about a month, a reminder was sent to the representatives of the federations, asking them to solicit the participation of their members again. The overall data collection took two months and 259 Italian clown therapists participated, including 69 males (27%) and 190 females (73%). The mean age was 40 years (SD = 11.84) with a range of 18 to 72 years. 

As the current total estimate of the Italian clown therapists is about 3000, the group of study participants represented approximately 7% of the reference population. 

### 2.2. Tools

The questionnaire consisted of a socio-demographic section and three main instruments:

(1) *Volunteer Self-Efficacy Scale* (SAV) [49]. This scale includes 19 items, rated on a 5-point Likert scale. It measures the degree of self-efficacy perceived by the subject, relating to four areas:

- The ability to express and share positive emotions and to manage negative ones, overcoming difficulties and moments of stress and tension.
- Empathy and, therefore, the ability to recognize in others, through the mechanism of identification, feelings and moods.
- Personal competence in voluntary work, such as the ability to successfully carry out one’s task, to learn and use the techniques necessary to be an effective support to people.
- Collective competence, such as coordinating with other association members in managing activities, and the ability to manage relationships with others. Reliability for this study showed an overall Cronbach’s alpha value of 0.88, CI 95% [0.86–0.90] and McDonald’s omega of 0.89, CI 95% [0.86–0.90].

(2) *Volunteer Process Model* (VPM) [48,50]. The scale consists of 30 items, rated on a 5-point Likert scale. Reliability for this study showed a Cronbach’s alpha value of 0.93, CI 95% [0.92–0.94] and McDonald’s omega of 0.93, CI 95% [0.92–0.95]. The tool measures six motivational functions highlighted by Omoto and Snyder, namely utilitarian, social, values, growth, knowledge, and protective functions:

- Values functions are linked to the subject’s value system and, in particular, to value instances, the implementation of pro-social and altruistic behaviors. The volunteer places a positive value on the well-being of others and, at the same time, uses his/her image as a volunteer to support the idea of a positive self and his/her own value system. Reliability for this study showed a Cronbach’s alpha value of 0.70 CI 95% [0.62–0.77] and McDonald’s omega of 0.71 CI 95% [0.65–0.78].
- Knowledge/skills function, i.e., through volunteering, the person can acquire new knowledge and skills or improve their previous ones. Reliability for this study showed a Cronbach’s alpha value of 0.75 CI 95% [0.70–0.79] and McDonald’s omega of 0.76 CI 95% [0.71–0.80].
- Social function (relational aspect), i.e., through volunteering, the subject can increase his friendships and knowledge, creating new relationships and satisfying his needs by belonging to an organized group of volunteers. Another motivation which performs this social function is that which drives the subject to become a volunteer to join socially desirable groups. Reliability for this study showed a Cronbach’s alpha value of 0.78 CI 95% [0.74–0.82] and McDonald’s omega of 0.79 CI 95% [0.74–0.83].
- Protective function, i.e., through the performance of a volunteer activity, the individual seeks to reduce the sense of guilt for being more fortunate than others. Another motivation that serves as protective concerns the possibility for the individual to focus his attention on helping people, thus moving away from their problems. Similarly, therefore, the confirmation of one’s skills and abilities, which the subject receives as a result of his helping activities, will allow him to face internal conflicts with greater self-awareness, managing anxieties and uncertainties on the basis of the value attributed to his efficiency. Reliability for this study showed a Cronbach’s alpha value of 0.77 CI 95% [0.73–0.81] and McDonald’s omega of 0.80 CI 95% [0.76–0.84].
- Growth-self-enhancement function, i.e., the motivations of the subject underlying the need/desire to increase his self-esteem, reinforcing a positive self-image through his prosocial behavior. Reliability for this study showed a Cronbach’s alpha value of 0.80 CI 95% [0.76–0.84] and McDonald’s omega of 0.82 CI 95% [0.78–0.85].
- Utilitarian function, i.e., the subject’s desire to improve his professional skills through volunteer activities, particularly for career development and professional curriculum. Reliability for this study showed a Cronbach’s alpha value of 0.84 CI 95% [0.81–0.87] and McDonald’s omega of 0.84 CI 95% [0.81–0.87].

(3) *Italy Personality Inventory* (Itapi*-G*) [47 The instrument consists of 105 items with a Likert scale ranging from 1 (strongly disagree) to 4 (strongly agree) and measures seven factors-traits which are:

- Dynamism (initiative, curiosity, liveliness), reliability for this study showed a Cronbach’s alpha value of 0.70 CI 95% [0.64–0.76] and McDonald’s omega of 0.70 CI 95% [0.64–0.76].
- Vulnerability (discomfort, fear, suffering), reliability for this study showed a Cronbach’s alpha value of 0.77 CI 95% [0.72–0.81] and McDonald’s omega of 0.78 CI 95% [0.73–0.82].
- Empathy (solidarity, sociability, sensitivity), reliability for this study showed a Cronbach’s alpha value of 0.69 CI 95% [0.63–0.73] and McDonald’s omega of 0.70 CI 95% [0.64–0.75].
- Conscientiousness (perseverance, precision, rationality), reliability for this study showed a Cronbach’s alpha value of 0.75 CI 95% [0.67–0.82] and McDonald’s omega 0.77 CI 95% [0.70–0.84].
- Imagination (creativity, feeling, fantasy), reliability for this study showed a Cronbach’s alpha value of 0.75 CI 95% [0.70–0.80] and McDonald’s omega of 0.77 CI 95% [0.73–0.82].
- Defensiveness (distrust, rigidity, materiality), reliability for this study showed a Cronbach’s alpha value of 0.70 CI 95% [0.64–0.79] and McDonald’s omega of 0.71 CI 95% [0.65–0.81].
- Introversion (introspection, self-sufficiency, isolation), reliability for this study showed a Cronbach’s alpha value of 0.73 CI 95% [0.67–0.78] and McDonald’s omega of 0.73 CI 95% [0.67–0.78].

### 2.3. Statistical Analysis

Descriptive analyses (percentages, means, standard deviation, skewness and kurtosis, confidence intervals); *t*-test for comparison of scores with respect to gender; Pearson’s bivariate correlations; testing of univariate and multivariate regression assumptions; hierarchical regression.

## 3. Results

### Descriptive Analyses

Table 1 and Table 2 below show the descriptive statistics of socio-demographic variables of the sample and measures of perceived self-efficacy, motivation, work experience and trait dispositions, respectively. 

For participants, the motivation factors with higher mean scores were found to be: values (M = 3.61 SD = 0.55), self-enhancement (M = 3.60 SD = 0.73), and knowledge/skills (M = 3.48 SD = 0.69). This result shows that needs attributable to the internal sphere of the person are higher in clown therapists: acting according to higher instances of values whose importance is internally felt, desiring growth and self-improvement, constantly seeking to acquire additional knowledge and skills. In contrast, the motivational factors with lower mean scores are all oriented to the sphere outside the person: deriving opportunities for concrete utility, obtaining protection from the group or institution, acquiring opportunities for new relationships and social contacts. In terms of gender differences, females in the sample showed significantly higher scores for the purposes of self-enhancement (t = −2.239; *p* = 0.026 CI 95% −0.429; −0.027), knowledge/skills (t = −2.212; *p* = 0.028 CI 95% −0.405; −0.023), and values (t = −2.472; *p* = 0.014 CI 95% −0.340; −0.038), demonstrating a greater intensity of the drive from internal motivational factors. The average level of perceived self-efficacy of the sample reports a score in the medium/high range (M = 3.63 SD = 0.48), indicating that overall, the clown therapists who participated in the study had a good perception of appropriateness and competence in the role. There were no significant differences in perceived self-efficacy with respect to gender, educational qualification, marital status, having had specific training prior to undertaking this activity, or target population (children, disabled, psychiatric patients, the elderly). 

Regarding the trait measure carried out by ITAPI-G, the factors with the highest mean scores were empathy (M = 3.38 SD = 0.41), dynamism (M = 3.13 SD = 0.50), imagination (M = 3.34 SD = 0.53) and conscientiousness (M = 2.95 SD = 0.55). The prevailing profile shows a clown therapist with pronounced active and creative empathic resonance, but at the same time with a disposition to control and good planning of actions taken. On the opposite polarity is recorded a profile substantially less suited to the delicate task of hospital animation, which exhibits traits of vulnerability (M = 2.28 SD = 0.63), introversion (M = 2.34 SD = 0.63) and defensiveness (M = 2.54 SD = 0.47). Excessive emotional sensitivity and vulnerability, coupled with defensive closure, clearly prevent the caregiver with these characteristics from being able to establish an effective and collaborative helping relationship with the team, but more importantly, they expose the person to absorbing and increasing levels of stress and tension that are not effectively modulated or regulated. Table 3 below illustrates the associations among the variables that emerged in the study conducted.

As shown in Table 3, perceived self-efficacy correlated most strongly with value-oriented motivation (0.360 **), knowledge/skills search (0.284 **), self-enhancement orientation (0.207 **), sociality need (0.190 **), while among the dispositional factors with the strongest strength of association were dynamism (0.349 **), empathy (0.232 **), conscientiousness (0.207 **), imagination (0.198 **), and in inverse association, vulnerability (−0.213 **) and introversion (−0.187 **). This set of correlations shows that a good level of perceived self-efficacy in the clown therapist is generally associated with the gratification of needs of the person’s inner sphere (value orientation, knowledge acquisition, self-improvement) and also with the pursuit of social ties and contacts. In terms of traits, the importance of the capacity for activation, positive and constructive adaptation to situations, the ability to establish a relationship of empathetic openness and attunement with the other, without prejudice and defensive closures, is emphasized. Equally interesting are the correlations between individual motivations and personality traits of the clown therapist. Personal utility research showed significant associations with vulnerability (0.220 **), experience (−0.195 **) and imagination (0.178 **). In this case, it is likely that the awareness of personal fragility is accompanied by the desire for compensation by obtaining concrete achievements, without having a specific plan but rather accompanying the path with fantasies and aspirations cultivated in the imagination. The negative association with experience testifies that as practice in clown therapy continues, the driving factor is no longer that of an expectation of personal usefulness, instead other factors evidently gain full prevalence.

The self-improvement motive showed major correlations with empathy (0.279 **), imagination (0.252 **), defensiveness (0.213 **), vulnerability (0.191 **), and conscientiousness (0.181 **). Clown therapists with greater interpersonal skills and who are more prone to emotional sharing are also those who seek further growth through direct contact with a suffering humanity to be sustained through the use of creative means and diffusion into alternative and fantastic scenarios. The association with conscientiousness indicates the seriousness of the task undertaken and the awareness of having to continually improve in perfecting the means and techniques employed. The presence of associations with defensiveness and vulnerability could refer to the desire for more fragile individuals to measure themselves against this delicate task of hospital animation, believing that they can thereby work indirectly in favor of overcoming their perceived vulnerabilities.

The need for protection showed the main correlations with empathy (0.292 **), defensiveness (0.258 **), vulnerability (0.234 **), imagination (0.212 **), and experience (−0.193 **). Again, individuals with frailty and compensatory defensive set-ups can imagine their belonging to the clown therapeutic animation group a sense of protection, identifying themselves in a positive, meaningful, and beneficial role for other, less fortunate people. The inverse association with experience shows that over time the motivation for surrogate protection entrusted to others can give way to a greater awareness of self and one’s autonomy.

The need for sociability reported the strongest correlations with empathy (0.285 **), imagination (0.225 **) and inverse with experience (−0.166 **). Motivation characterized by the search for social contact naturally showed a priority connection with empathic disposition but also with a productive effervescence of mind capable of creative outbursts and brilliant inventiveness. The inverse correlation with experience may indicate that over time this initial need for externalized orientation (bonding and social contact) is not as strong, while at the same time, as noted in previous comments, intrinsic motivational factors of the person may gain more prominence.

Motivation oriented toward acquiring knowledge and skills showed correlations with the trait of dynamism (0.255 **), imagination (0.246 **), empathy (0.223 **), experience (−0.201 **), and vulnerability (0.176 **). Plausibly, a dynamic and mentally active profile is driven by a lively intellectual curiosity and aspiration to acquire further skills. Those who show greater openness to relationships and disposition to mutual understanding are also more oriented toward gathering new knowledge and enhancing their skills precisely through interactive exchange. Those who feel more of their own limitations in terms of personal vulnerability may feel the need to engage in new activities to curb these gaps in their wealth of experience. 

Finally, the clown therapist’s value motivation reported significant correlations with empathy (0.381 **), imagination (0.260 **), dynamism (0.258 **), introversion (−0.214 **), and experience (−0.164 **). Once again, an active, open, and dynamic profile appears to be a favorable disposition for an agenticity guided by higher and general value instances, not confined to the mere sphere of self-interest. Introversion, on the other hand, appears more as rigid fixation on one’s ego that renders the person incapable of acting in a genuinely prosocial manner. 

The inverse associations with experience, found on as many as five of the six motivational functions, could also have a different interpretation from the one presented earlier: it could be a sign of a decreased involvement of the practitioners who over time may begin to perceive a certain disillusionment from previous expectations, a motivational decline that may sooner or later also lead them to leave the clown therapist activity.

In order to identify predictors of perceived practitioner self-efficacy, a hierarchical regression was performed on motivational and trait variables. The preliminary verifications of the regression assumptions excluded the presence of multivariate outliers. Mardia’s multivariate kurtosis index (245.161) was in fact below the critical value [p (p + 2) = 255]; therefore, the relationship between the variables can be considered substantially linear. Low co-linearity was indicated by the low variance inflation factor (VIF) values < 2 and high tolerance values > 0.60. For verification of the assumptions on the residuals, the average between the standardized and raw residuals was equal to 0; the Durbin–Watson test had a value of 1.88 and was therefore indicative of the absence of autocorrelation. 

A hierarchical multiple regression was run to determine if the addition of values, dynamism, vulnerability, conscientiousness, and experience improved the prediction of the perceived self-efficacy. The full model resulted statistically significant, R^2^ = 0.305, F(5,258) = 22.206, *p* < 0.001; adjusted R^2^ = 0.291. The regression model included values and dynamism at step 1, vulnerability at step 2, conscientiousness at step 3, and experience at step 4. The results of the hierarchical multiple linear regressions are presented in Table 4.

In the regression model, with self-efficacy as outcome variable, values and dynamism jointly explained a 20% portion of the outcome variability. Adding vulnerability at the second step provided a significant improvement in the explained variance, which reached 24%. By adding conscientiousness at the third step, the explained variance further significantly increased to 28%. By adding experience at the fourth step, the explained variance further significantly increased to 31%. Standardized beta values were significant, with a positive sign for values, dynamism, conscientiousness, experience, and a negative sign for vulnerability. 

Two additional analyses were then conducted using the selection variable in order to compare: 

(1) The predictive pattern of self-efficacy in those working primarily with children (n = 195) compared with those working primarily with the elderly (n = 58). In fact, these represented the most frequent targets, compared with clown therapists working with the disabled (N = 4) and psychiatric patients (N = 2).

(2) The predictive model of self-efficacy in those who work mainly in pairs (N = 111) compared to clown therapists who usually work in teams (N = 144).

Considering the group of clown therapists who work with children, the statistically significant full model was the following: R^2^ = 0.315, F(5,194) = 17.371, *p* < 0.001; adjusted R^2^ = 0.297. The regression model included values and dynamism at step 1, vulnerability at step 2, experience at step 3, and conscientiousness at step 4. The results of the hierarchical multiple linear regressions are presented in the following Table 5. 

In the regression model, with self-efficacy as outcome variable, values and dynamism jointly explained a 22% portion of the outcome variability. Adding vulnerability at the second step provided a significant improvement in the explained variance, which reached 27%. By adding experience at the third step, the explained variance further significantly increased to 30%. By adding conscientiousness at the fourth step, the explained variance further significantly increased to 31%. Standardized beta values were significant, with a positive sign for values, dynamism, conscientiousness, experience, and a negative sign for vulnerability.

Considering, instead, the group of clown therapists who work with the elderly, the statistically significant full model was the following: R^2^ = 0.303, F(2,57) = 11.952, *p* < 0.001; adjusted R^2^ = 0.278. The results of the hierarchical multiple linear regressions are presented in following Table 6. In the regression model, with self-efficacy as outcome variable, conscientiousness and experience jointly explained a 30% portion of the outcome variability. 

Considering the group of clown therapists who routinely work in pairs, the statistically significant full model was the following: R^2^ = 0.332, F(4,110) = 13.197, *p* < 0.001; adjusted R^2^ = 0.307. The regression model included values and vulnerability at step 1, dynamism at step 2, and experience at step 3. The results of the hierarchical multiple linear regressions are presented in Table 7. In the regression model, with self-efficacy as outcome variable, values and vulnerability jointly explained a 26% portion of the outcome variability. Adding dynamism at the second step provided a significant improvement in the explained variance, which reached 29%. By adding experience at the third step, the explained variance further significantly increased to 33%. Standardized beta values were significant, with a positive sign for values, dynamism, experience, and a negative sign for vulnerability. 

Considering the group of clown therapists who routinely work as a team, the statistically significant full model was the following: R^2^ = 0.302, F(4,143) = 15.069, *p* < 0.001; adjusted R^2^ = 0.282. The regression model included values and dynamism at step 1, conscientiousness at step 2, and vulnerability at step 3. The results of the hierarchical multiple linear regressions are presented in Table 8. 

In the regression model, with self-efficacy as outcome variable, values and dynamism jointly explained a 23% portion of the outcome variability. Adding conscientiousness at the second step provided a significant improvement in the explained variance, which reached 27%. By adding vulnerability at the third step, the explained variance further significantly increased to 30%. Standardized beta values were significant, with a positive sign for values, dynamism, conscientiousness, and a negative sign for vulnerability. 

## 4. Discussion

The present study aimed to identify the main predictors of perceived self-efficacy in a sample of Italian volunteer clown therapists. The hypothesis was that these predictors were to be identified among the individual motivations, the dispositional trait features, and considering specific situational aspects. Similar to previous studies [29], the sample was characterized by a medium-high level of education; in fact, about half had a university degree; almost all had had specific training in clown therapy before undertaking the activity and belonged to associations with a number of members greater than 20. Most worked with hospitalized children.

Descriptive analyses showed that the motivational factors that reported the highest scores were: the values dimension linked to the value system of the subject and, in particular to value-related instances, to the implementation of pro-social and altruistic behavior, giving a positive value to the welfare of others and, at the same time, exploiting one’s image supporting the idea of a positive self and one’s value system; the desire for personal growth that is, the need/desire to increase one’s self-confidence, reinforcing the positive image of oneself through one’s prosocial behavior, the opportunity to acquire new knowledge and skills or improve previous ones. In particular, these three motivational factors were more evident in the group of females. 

The motivational factors with the lowest scores were the utility function, i.e., the desire to improve one’s professional skills through voluntary activities, particularly for career development and professional curriculum, and the protective function, that is, the possibility for the subject to focus his attention on helping people, thus moving away from personal problems. An intermediate score level reported the social function, or relational aspect of the work, which allows the individual to increase his friendships and knowledge, creating new relationships and satisfying his needs through his affiliate membership of an organized group.

The hierarchical regression model on self-efficacy, which included both the motivational and dispositional elements, which together with self-efficacy had higher values of significant correlations, confirmed value attribution as the only incident motivational factor, and with higher weight than the three significant dispositional elements, namely the dynamic personality trait, conscientiousness and low vulnerability of the person. Among the situational and socio-demographic characterization variables, the clown therapist’s experience was significant, and therefore this variable was also included within the overall model. 

The values aspect is a very important component. The operator will feel more capable to the extent that he perceives that what he is doing is coherent with the basic values in which he personally believes and that the environment he is in contact with (family, friends, community) has transmitted to him: that is, to give support and comfort to others who are in a condition of particular need, first of all to children to feel emotional involvement and natural enthusiasm towards other people in difficulty and to firmly believe in the importance of helping. These aspects that have emerged are congruent with what has been shown by previous work [51,52,53]. The issue of clowning as an ethical care practice has been well illustrated in Molterer et al. [54]. The incorporation of values into the identity representation of the clown therapist also finds correspondence in the work of Meier and Andritsou [55]. 

Among the dispositional aspects, the dynamism of the person is another significant characteristic that indicates very important qualities for the activity that the clown therapist is called to perform: resourcefulness, curiosity, vivacity. An enterprising person is persuasive, assertive, confident, energetic, optimistic, adventurous, sociable. The curious person is eager to know, to learn, loves challenges, eschews prejudices and stereotypes. Liveliness indicates an exuberant vitality or even intellectual readiness and versatility. All these characteristics of a person’s dynamic traits are a fundamental prerequisite for the clown therapist’s creativity in order to improvise according to what is found in the healthcare setting, to be more cheerful, less serious, and be able to produce a higher quantity of humor content. Already in Dionigi’s studies [30,32] the positive synergy for such activities of personal traits, such as openness to experience, extraversion, and agreeableness, has been highlighted, while more recently Ruch et al. have emphasized the importance of cheerfulness, suggesting five relationships between trait cheerfulness and a cheerful state: high trait cheerful individuals have a lower threshold, a higher intensity, a longer duration, a higher robustness of cheerful mood (even when facing adversity), and a faster mood recovery (after a mood alteration towards the negative) [56]. 

The third predictor is low vulnerability, that is, the absence of emotional fragility and high sensitivity, the ability to have control over emotions and the ability to return to the basic emotional state. It is important to be able to manage one’s own fears and not have important reasons for individual suffering in order to be able to appropriately mitigate experiences of human contact in highly problematic contexts, such as hospital wards for children, facilities for the disabled, psychiatric patients and the elderly. Some scholars have stressed the risk of emotional exhaustion for clown therapists and the need to have appropriate coping strategies to compensate for their vulnerabilities [57]. Dionigi had shown that the best predictor to keep in mind for emotional exhaustion in clown therapists was the neuroticism trait [58]. Some have suggested it can limit the risk of emotional exhaustion, by learning to keep the role being played separate from one’s own person [59,60,61], while others recommend a number of expedients that can be applied to mitigate the risk of burnout: spacing out vacations, frequently consulting with a supervisor, and relying on consultation with a mental health professional in the event of perceived discomfort [62,63,64]. 

The fourth predictor is conscientiousness, which is the tendency to perform assigned tasks well and to take obligations to others seriously. Conscientious people tend to be efficient and organized, rather than accommodating and disorderly. Clowns are required to attend rigorous training before entering the health setting and must be aware of their role as well as of the risks related to a wrong approach to patients or to a lack of competence, as conscientious individuals are able to manage emotions, be self-disciplined, and focus on goal achievement. This trait element in the profile of the clown therapist has already been highlighted in the previous work by Dionigi [32]. In line with other research on volunteers in the hospital setting, a higher score was found on the traits of conscientiousness and openness, thereby indicating a propensity for direct and authentic relationship and human contact but at the same time awareness of the importance of the task and role played [65]. 

The fifth and final predictor of self-efficacy included in the model was experience. Here come into play over time both the subjective and collective aspect of the wealth of well-handled situations, of difficulties which have been overcome, of techniques learned and improved, but also of feedback received, of models imitated, of comparisons with more experienced colleagues, of sharing a certain culture of animation, of strengthening the sense of belonging to one’s own group/association, the awareness of a role that over time is consolidated and finds wide appreciation even outside one’s own environment. As illustrated in other studies, over time experiences help shape the role identity of the hospital volunteer and define any variations in the “salience” of the role, which in turn contribute significantly to the intention to retain or leave the activity [66,67,68].

By comparing the predictive model of the group working prevalently with children with that of the group working with the elderly, it was observed that in the second case the predictive significance was limited to the variables of conscientiousness and experience, while it was less for those of dynamism, low vulnerability, and value. This may mean that, in order to create professional satisfaction in the operator, intervention with the elderly requires greater technical awareness, careful planning and targeting of interventions, familiarity with age-specific pathologies (dementia, depression) and selection over time of effective communication strategies to stimulate the elderly’s mood, cognitive skills and alleviate their sense of dependence and loneliness. With regard to the delicate and relevant function of clown therapy with the elderly, indications that are congruent with our results can be found in several studies that emphasize the desirability of practitioners who have received adequate professional training in the administration of clown therapy sessions aimed at soliciting multiple benefits, even in the presence of the neurodegenerative pathologies typical of aging [69,70,71,72].

Whereas regarding work with children, greater importance is given to the emotional impact and the empathic resonance of the setting (for both operators and children), the operator’s dynamic and creative ability to engage and defocus the children, thus triggering positive emotions, imagination, and identification in them, demonstrating at the same time excellent coping skills and personal resilience, nourished by confidence in the human and social value of the work carried out. With regard to the benefits of professional clowning with children having problems of varying severity, recurrent and congruent indications can be found in recent literature [73,74,75,76,77,78].

A final comparison in the study was made between the predictive model of the group working predominantly in pairs versus those working predominantly in teams. In the first case, the most incisive predictors were value motivation and low vulnerability of the operator; the conscientiousness variable was not significant. This could highlight the specific nature of the work of a pair, where synergy, complicity, harmony, and mutual support of the members in the face of possible difficulties become important for satisfaction. The emotional fragility of one of the members, more than their organization and technical preparation, can have a serious impact on the final result; moreover, the weakening over time of the (social as well as humanitarian) value attributed to their interventions can affect the harmony of the pair of operators. The necessary integration and complementarity of clowns working in pairs was addressed by Dionigi [5], quoting Flangini [79] in turn: “The clown doctors usually work in pairs: one performer plays the role of the “white clown” who represents the rational voice of reason and the orderly decision maker. His partner plays the role of emotional character who is also the problem maker. Each person has his own style, set of practices, comic gags, gestures, vocabulary, and voice”. With reference to the delicate relational and communicative synchronization of the affective mirroring functions of the two clowns, an interesting description can be found in Linge’s contribution [80], where it is shown how children’s mirroring takes place in an external scenario where the two clowns assume two opposite positions; this allows the child to see his/her own inner problems at a distance and to approach this inner world at his/her own, often calmer, pace. This triangular process ensures that the clowns convey the message to the child in a more digestible, humoristic form. Given the delicacy of such interventions, the acquisition of full competence and mastery in the use of resources and modulation of expressive means appears to be an indispensable professional requirement that implies adequate training and careful evaluation through expert supervision [81,82,83,84].

In team operators, satisfaction derives from the dynamic capacity expressed by the individual in the group, from the recognition of competence that the group refers to, and from the sharing of the value scenario that animates the intervention. Experience is not significant in the team model because the group can compensate and support in the activities, even the youngest and most inexperienced members, without this jeopardizing the achievement of objectives. In the literature, there appears to be an increasingly evident need for clowns to be able to collaborate and integrate into the multidisciplinary health care unit so that the benefits of their intervention are maximized on multiple levels, for child patients, parents, physicians, and the staff of health care professionals, such as nurses, physical therapists, and psychologists. In this regard, it becomes important to train new clown therapists to fully integrate and collaborate with both their team and other professionals in the hospital setting [1,85,86,87,88].

## 5. Practical Implications of the Study Results

The identification of both the positive and negative predictors of the clown therapist’s perceived self-efficacy offers important pointers to consider in the orientation, selection, and training of aspiring new clown therapy practitioners. Since perceived self-efficacy is in turn a predictor of retention, in order to limit instances of dropouts that may be determined over time by various factors, such as dissatisfaction, frustration, burn-out, demotivation, etc., a careful preliminary assessment should be made by considering the risk of trait elements that are not conducive to performing the delicate activity of hospital animation, such as introversion, vulnerability, defensiveness, or certain motivational aspects oriented mainly by pragmatic reasons and the search for gratification of the needs of one’s own person, rather than aimed at intervening in favor of the needs of others. Fostering retention and limiting drop-out is important, as excessive operator turnover prevents the maturation of expertise and the integrated and stabilized construction of cohesive and effective work teams. The knowledge of positive predictors for the gratified performance of hospital clowning actions indicates qualities and aspects of the person that are not only to be sought but also enhanced through training, updating, and expert supervision actions. The differentiations illustrated in the results and related to the work targets (children, the elderly, the disabled) reiterate the need to acquire adequate and specialized technical and professional training with respect to the needs and characteristics of the target group. The results also show that the ability to synchronize in pair work will have to be particularly sought after and enhanced, with regard especially to the ability to collaborate and dispose oneself with an open attitude to confrontation and integration of the different professional cultures that make up the health care unit: physicians, nurses, technical assistants, other therapists, administrative staff, and patients’ families.

Of course, the results obtained through this study should also be considered in light of certain limitations. It is worth mentioning that this study was conducted on a sample of Italian clown therapists, and further research is needed to confirm these results for other cultures and nationalities. The relatively small sample further limits the findings, and replications on larger samples are required. The cross-sectional nature of the study should be supplemented with a future longitudinal design that can monitor changes in variables over a significant interval of activity performed by clowning practitioners in hospitals.

## 6. Conclusions

The results of the study largely confirmed the main hypotheses regarding the positive and negative predictors, although some differences from what was expected were found. The study first showed that the value orientation of clown therapists influenced the level of perceived self-efficacy, that is, the pursuit of actions with high social meaning, rather than orientation to situations and experiences that allow them to expand their knowledge and promote their person. Profiles with higher perceived self-efficacy were associated with the trait of dynamism and conscientiousness, whereas personal vulnerability was found to be a significant negative predictor of self-efficacy. A final significant predictor was that of experience, which includes both the subjective and collective aspect, including well-handled situations, feedback received, models imitated, comparisons with more experienced colleagues, the sense of belonging to one’s own group/association, and the awareness of a role that over time has been consolidated and is widely appreciated even outside one’s own environment. In contrast to expectations, among the positive predictors hypothesized, empathic and imagination disposition did not report significant roles in the regression model; nor did introversion and defensiveness disposition among the negative predictors; whereas, among the six motivational factors, only the clown therapist’s value drive proved to play a significant role in predicting perceived self-efficacy. With regard to the sub-hypotheses, for those who worked with children, the predictors identified in the general model of the study’s main hypotheses were confirmed; while with regard to the factorial weight, the value orientation was found to have greater weight in the clown therapy practitioner than the dynamic trait, contrary to what we had hypothesized. Even in the case of the sub-hypothesis concerning clown therapists engaged predominantly with a target group of elderly people, the results of the related regression showed dissimilarity to what was expected. In fact, instead of the predictor empathy, which was not met, the dispositional traits of conscientiousness and experience proved to be significant. The last sub-hypothesis, by which the clowning intervention conducted in pairs was differentiated from an intervention coordinated at the team level, showed a confirmation of the main predictors of self-efficacy: dynamism in pair work and conscientiousness as a prevalent and functional factor for synergistic integration in the team.

## Figures and Tables

**Table 1:** Descriptive statistics for socio-demographic variables.

Variables		N	(%)
**Gender**	Males	69	27.0
	Females	190	73.0
**Age**	18–72 years	259	100.0
**Status**	Single	80	30.9
	Fiancé	51	19.7
	Cohabitant	40	15.4
	Married	74	28.6
	Separated or divorced	14	5.4
**Children**	Yes	82	31.7
	No	177	68.3
**Education**	High school diploma	131	50.6
	Bachelor’s degree	58	22.4
	Master’s degree	52	20.1
	Postgraduate degree	18	6.9
**Experience**	Less than a year	12	4.6
	One year to five years	127	49.0
	Five to ten years	69	26.6
	More than ten years	51	19.7
**Specific training**	Yes	242	93.4
	No	17	6.6
**Target audience**	Children	195	75.3
	Disabled	4	1.5
	Psychiatric patients	2	0.8
	Elderly	58	22.4
**Mode of conducting**	Alone	4	1.5
	In pairs	111	42.9
	In a team	144	55.6
**Numerosity of the associations**	Less than ten members	8	3.1
	10 to 20 members	25	9.7
	More than 20 members	226	87.3

**Table 2:** Descriptive statistics for self-efficacy, motivations, work experience, and trait dispositions.

	M	SD	Ra	SK	SE	KU	SE	CI 95%
Self-Efficacy (SAV)	3.63	0.484	1–5	−0.001	0.151	−0.086	0.302	[3.57–3.69]
Utility (VPM)	2.16	0.804	1–5	0.569	0.151	0.090	0.302	[2.06–2.26]
Self-Enhancement (VPM)	3.60	0.732	1–5	−0.006	0.151	−0.699	0.302	[3.52–3.70]
Protection (VPM)	2.85	0.791	1–5	0.482	0.151	−0.048	0.302	[2.76–2.95]
Sociality (VPM)	3.17	0.803	1–5	−0.315	0.151	0.160	0.302	[3.08–3.27]
Knowledge/Skills (VPM)	3.48	0.695	1–5	−0.142	0.151	−0.006	0.302	[3.40–3.57]
Values (VPM)	3.61	0.551	1–5	−0.026	0.151	−0.371	0.302	[3.54–3.68]
Experience (years)	4.31	2.85	0–12	0.378	0.151	−0.860	0.302	[3.12–4.87]
Dynamism (ITAPI-G)	3.13	0.499	1–4	−0.478	0.151	0.766	0.302	[3.07–3.20]
Vunerability (ITAPI-G)	2.28	0.634	1–4	0.204	0.151	−0.212	0.302	[2.20–2.36]
Empathy (ITAPI-G)	3.38	0.414	1–4	−0.295	0.151	−0.526	0.302	[3.34–3.44]
Conscientiousness (ITAPI-G)	2.95	0.550	1–4	−0.163	0.151	−0.345	0.302	[2.89–3.03]
Imagination (ITAPI-G)	3.24	0.532	1–4	−0.755	0.151	0.851	0.302	[3.18–3.31]
Defensiveness (ITAPI-G)	2.54	0.467	1–4	−0.055	0.151	−0.188	0.302	[2.48–2.59]
Introversion (ITAPI-G)	2.34	0.628	1–4	0.168	0.151	−0.129	0.302	[2.27–2.42]

N = 259. SD = Standard Deviation; RA = Range; SK = Skewness; SE = Standard Error; KU = Kurtosis; CI = Confidence Interval.

**Table 3:** Bivariate correlations.

Self-Efficacy														
Utility	1													
Self-Enhancement	0.459 **	1												
Protection	0.513 **	0.672 **	1											
Sociality	0.532 **	0.582 **	0.673 **	1										
Knowledge/Skills	0.572 **	0.631 **	0.573 **	0.653 **	1									
Values	0.398 **	0.570 **	0.555 **	0.669 **	0.619 **	1								
Dynamism	0.349 **	0.147 *	0.155 *	0.126 *	0.155 *	0.255 **	0.258 **	1						
Vulnerability	−0.213 **	0.220 **	0.191 **	0.234 **	0.157 *	0.176 **	0.092	−0.135 *	1					
Empathy	0.232 **	0.070	0.279 **	0.292 **	0.285 **	0.223 **	0.381 **	0.301 **	−0.077	1				
Conscientiousness	0.207 **	−0.024	0.181 **	0.108	0.054	0.070	0.046	0.125 *	0.136 *	0.193 **	1			
Immagination	0.198 **	0.178 **	0.252 **	0.212 **	0.225 **	0.246 **	0.260 **	0.494 **	0.042	0.436 **	0.138 *	1		
Defensiveness	0.088	0.119	0.213 **	0.258 **	0.108	0.091	0.108	−0.026	0.334 **	0.007	0.352 **	0.051	1	
Introversion	−0.187 **	−0.077	−0.102	−0.015	−0.133 *	−0.101	−0.214 **	−0.094	0.309 **	−0.256 **	0.138 *	−0.114	0.248 **	1
Experience	0.113	−0.093	−0.195 **	−0.193 **	−0.166 **	−0.201 **	−0.164 **	−0.075	−0.103	−0.023	0.012	0.047	−0.090	−0.098

** Correlation is significant at the 0.01 level (two-tailed). * Correlation is significant at the 0.05 level (two-tailed).

**Table 4:** Results of Hierarchical Linear Regression Analyses with whole Sample.

Outcome Variable			
Perceived Self-Efficacy			
Independent Variable	Adjust. R^2^	∆R^2^	β
Step 1	0.194 ***	0.200 ***	
Values			0.289 ***
Dynamism			0.274 ***
Step 2	0.234 ***	0.043 ***	
Values			0.318 ***
Dynamism			0.238 ***
Vulnerability			−0.211 ***
Step 3	0.269 ***	0.038 ***	
Values			0.319 ***
Dynamism			0.209 ***
Vulnerability			−0.242 ***
Conscientiousness			0.199 ***
Step 4	0.291 ***	0.024 **	
Values			0.342 ***
Dynamism			0.218 ***
Vulnerability			−0.225 ***
Conscientiousness			0.193 **
Experience			0.159 **

Note: N = 259; β = standardized beta value. ** *p* < 0.01. *** *p* ≤ 0.001.

**Table 5:** Results of Hierarchical Linear Regression Analyses of Clown Therapists working with Children.

Outcome Variable			
Perceived Self-Efficacy			
Independent Variable	Adjust. R^2^	∆R^2^	β
Step 1	0.215 ***	0.223 ***	
Values			0.321 ***
Dynamism			0.268 ***
Step 2	0.260 ***	0.048 ***	
Values			0.348 ***
Dynamism			0.234 ***
Vulnerability			−0.223 ***
Step 3	0.284 **	0.027 **	
Values			0.381 ***
Dynamism			0.248 ***
Vulnerability			−0.200 **
Experience			0.171 **
Step 4	0.297 *	0.016 *	
Values			0.380 ***
Dynamism			0.231 ***
Vulnerability			−0.225 ***
Experience			0.165 **
Conscientiousness			0.129 *

Note: N = 195; β = standardized beta value. * *p* < 0.05 ** *p* < 0.01. *** *p* ≤ 0.001.

**Table 6:** Results of Hierarchical Linear Regression Analyses of Clown Therapists working with the Elderly.

Outcome Variable			
Perceived Self-Efficacy			
Independent Variable	Adjust. R^2^	∆R^2^	β
	0.278 ***		
Conscientiousness		0.237 ***	0.480 ***
Experience		0.066 *	0.257 *

Note: N = 58; β = standardized beta value. * *p* < 0.05 *** *p* ≤ 0.001.

**Table 7:** Results of Hierarchical Linear Regression Analyses of Clown Therapists working in Pairs.

Outcome Variable			
Perceived Self-Efficacy			
Independent Variable	Adjust. R^2^	∆R^2^	β
Step 1	0.243 ***	0.256 ***	
Values			0.375 ***
Vulnerability			−0.395 ***
Step 2	0.274 *	0.037 *	
Values			0.321 ***
Vulnerability			−0.354 ***
Dynamism			0.203 *
Step 3	0.307 *	0.038 *	
Values			0.330 ***
Vulnerability			−0.331 ***
Dynamism			0.220 *
Experience			0.198 *

Note: N = 111; β = standardized beta value. * *p* < 0.05. *** *p* ≤ 0.001.

**Table 8:** Results of Hierarchical Linear Regression Analyses of Clown Therapists working in Teams.

Outcome Variable			
Perceived Self-Efficacy			
Independent Variable	Adjust. R^2^	∆R^2^	β
Step 1	0.217 ***	0.228 ***	
Values			0.310 ***
Dynamism			0.294 ***
Step 2	0.250 **	0.037 **	
Values			0.301 ***
Dynamism			0.281 ***
Conscientiousness			0.193 **
Step 3	0.282 **	0.037 **	
Values			0.319 ***
Dynamism			0.248 **
Conscientiousness			0.239 **
Vulnerability			−0.200 **

Note: N = 144; β = standardized beta value. ** *p* < 0.01. *** *p* ≤ 0.001.

## Data Availability

The datasets analysed during the current study are available from the corresponding author on reasonable request.
